# (*Z*)-3-(9-Anthr­yl)-1-(2-thien­yl)prop-2-en-1-one^1^

**DOI:** 10.1107/S1600536809031900

**Published:** 2009-08-15

**Authors:** Hoong-Kun Fun, Thitipone Suwunwong, Nawong Boonnak, Suchada Chantrapromma

**Affiliations:** a X-ray Crystallography Unit, School of Physics, Universiti Sains Malaysia, 11800 USM, Penang, Malaysia; b Crystal Materials Research Unit, Department of Chemistry, Faculty of Science, Prince of Songkla University, Hat-Yai, Songkhla 90112, Thailand

## Abstract

There are two crystallographically independent mol­ecules in the asymmetric unit of the title heteroaryl chalcone, C_21_H_14_OS: the dihedral angle between the thio­phene and anthracene rings is 75.07 (17)° in one mol­ecule and 76.32 (17)° in the other. The crystal structure is consolidated by short C⋯O [3.348 (5)–3.394 (5) Å], C⋯S [3.607 (5)–3.666 (5) Å] and S⋯O [2.926 (3) Å] contacts, as well as by C—H⋯π and π–π inter­actions [*Cg*⋯*Cg* = 3.745 (3) Å].

## Related literature

For related structures, see: Chantrapromma *et al.* (2009 ▶); Suwunwong *et al.* (2009*a* ▶,*b* ▶). For background to and applications of chalcones, see: Oliveira *et al.* (2007 ▶); Patil & Dharmaprakash (2008 ▶); Saydam *et al.* (2003 ▶); Svetlichny *et al.* (2007 ▶). For the stability of the temperature controller used in the data collection, see Cosier & Glazer, (1986 ▶).
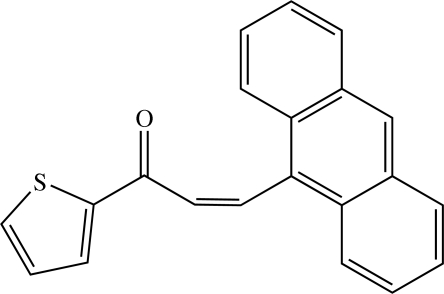


## Experimental

### 

#### Crystal data

C_21_H_14_OS
*M* *_r_* = 314.39
Orthorhombic, 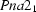

*a* = 14.6675 (2) Å
*b* = 5.5096 (1) Å
*c* = 37.9823 (4) Å
*V* = 3069.43 (8) Å^3^
*Z* = 8
Mo *K*α radiation
μ = 0.21 mm^−1^
*T* = 100 K
0.30 × 0.12 × 0.10 mm

#### Data collection

Bruker APEXII CCD area-detector diffractometer
Absorption correction: multi-scan (*SADABS*; Bruker, 2005 ▶) *T*_min_ = 0.939, *T*_max_ = 0.979
28929 measured reflections
6662 independent reflections
5348 reflections with *I* > 2σ(*I*)
*R*_int_ = 0.055

#### Refinement

*R*[*F* ^2^ > 2σ(*F* ^2^)] = 0.065
*wR*(*F* ^2^) = 0.200
*S* = 1.06
6662 reflections
391 parameters
1 restraint
H-atom parameters constrained
Δρ_max_ = 1.58 e Å^−3^
Δρ_min_ = −0.82 e Å^−3^
Absolute structure: Flack (1983 ▶), 3093 Friedel pairs
Flack parameter: 0.09 (15)

### 

Data collection: *APEX2* (Bruker, 2005 ▶); cell refinement: *SAINT* (Bruker, 2005 ▶); data reduction: *SAINT*; program(s) used to solve structure: *SHELXTL* (Sheldrick, 2008 ▶); program(s) used to refine structure: *SHELXTL*; molecular graphics: *SHELXTL*; software used to prepare material for publication: *SHELXTL* and *PLATON* (Spek, 2009 ▶).

## Supplementary Material

Crystal structure: contains datablocks global, I. DOI: 10.1107/S1600536809031900/tk2522sup1.cif

Structure factors: contains datablocks I. DOI: 10.1107/S1600536809031900/tk2522Isup2.hkl

Additional supplementary materials:  crystallographic information; 3D view; checkCIF report

## Figures and Tables

**Table 1:** Hydrogen-bond geometry (Å, °)

*D*—H⋯*A*	*D*—H	H⋯*A*	*D*⋯*A*	*D*—H⋯*A*
C3*A*—H3*AA*⋯*Cg*3^i^	0.93	2.99	3.679 (5)	132
C10*A*—H10*A*⋯*Cg*2^ii^	0.93	2.95	3.694 (5)	138
C10*B*—H10*B*⋯*Cg*5^i^	0.93	2.93	3.594 (5)	129
C15*A*—H15*A*⋯*Cg*3^iii^	0.93	2.76	3.550 (5)	143
C15*B*—H15*B*⋯*Cg*6^iii^	0.93	2.94	3.689 (5)	139
C19*A*—H19*A*⋯*Cg*4	0.93	2.72	3.486 (5)	140
C19*B*—H19*B*⋯*Cg*1^iii^	0.93	2.72	3.458 (5)	137

Symmetry codes: (i) 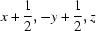; (ii) 
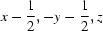
; (iii) 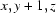. *Cg*1, *Cg*2, *Cg*3, *Cg*4, *Cg*5 and *Cg*6 are the centroids of the S1*A*/C18*A*–C21*A*, C1*A*–C6*A*, C8*A*–C13*A*, S1*B*/C18*B*–C21*B*, C1*B*–C6*B* and C8*B*–C13*B* rings, respectively.

**Fig. 1.**
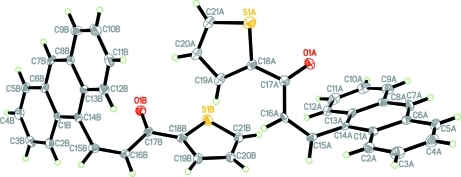
The molecular structure of (I), showing 50% probability displacement ellipsoids and the atom-numbering scheme.

## supplementary crystallographic information

### Comment

Chalcones have been studied for their chemical and biological activities for a long time. They have a wide range of applications such as in non-linear optical (NLO) materials (Patil & Dharmaprakash, 2008), fluorescent materials (Svetlichny *et al.*, 2007) and for showing various biological activities (Saydam *et al.*, 2003). The anthracene moieties are well known for their high absorption co-efficients as well as their high fluorescence yields (Oliveira *et al.*, 2007). These interesting properties has lead us to synthesize the title heteroaryl chalcone derivative, (I), which contains the donor sub-unit (anthracene) and fluorophore (thiophene) in order to study its NLO and fluorescent properties. We have previously synthesized and reported the crystal structures of chalcones and heteroaryl chalcone derivatives (Chantrapromma *et al.*, 2009; Suwunwong *et al.*, 2009*a*, *b*) which exist in the *E* configuartion. Herein, we report the crystal structure of the (I) which is in the *Z* configuration. Compound (I) crystallizes in the non-centrosymmetric orthorhombic space group *Pna*2_1_ and therefore, it should exhibit second-order nonlinear optical properties. Moreover, (I) also shows interesting fluorescence properties which will be reported elsewhere.

The asymmetric unit of (I) contains two molecules, *A* and *B*, with the same configuration but with slight differences in bond lengths and angles. The molecule of (I)(Fig. 1) exists in an *Z* configuration with respect to the C15=C16 double bond [1.360 (6) Å in molecule *A* and 1.331 (6) Å in molecule *B*]; the C14–C15–C16–C17 torsion angle = -3.7 (7)° in molecule *A* [-4.0 (7)° in molecule *B*]. The anthracene unit is essentially planar with the greatest deviation of 0.089 (5) Å at atom C11A [0.086 (5)Å at atom C3B]. The total molecule is twisted as the interplanar angle between thiophene and anthracene rings is 75.07 (17)° and the mean plane through the prop-2-en-1-one unit (C15–C17/O1) makes interplanar angles of 13.1 (3) and 71.2 (3)° with the thiophene and anthracene rings, respectively [the corresponding values are 76.32 (17), 15.2 (3) and 72.3 (3)° in molecule *B*]. The bond distances are comparable with related structures (Chantrapromma *et al.*, 2009; Suwunwong *et al.*, 2009*a*, *b*).

In the crystal packing, the molecules are connected by short C···O [3.348 (5)–3.394 (5) Å], C···S [3.607 (5)–3.666 (5) Å], and S···O [2.926 (3) Å] contacts. The crystal structure is further stabilized by C—H···π interactions (Table 1) and π–π interactions with the *Cg*_1_···*Cg*_4_^i^ distance being 3.745 (3) Å (i: 1/2 + *x*, -*y* + 1/2, *z*); *Cg*_1_ and *Cg*_4_ are the centroids of the S1A/C18A–C21A and S1B/C18B–C21B rings, respectively.

### Experimental

Compound (I) was synthesized by the condensation of anthracene-9-carbaldehyde (2 mmol, 0.41 g) with 2-acetylthiophene (2 mmol, 0.22 ml) in ethanol (30 ml) in the presence of NaOH (5 ml, 30 %). After stirring for 2 h, a yellow solid appeared which was then collected by filtration, washed with distilled water, dried and purified by repeated recrystallization using ethanol/acetone in a 1:5 ratio as solvent. Orange block-shaped crystals of (I) were obtained from hot ethanol by the slow evaporation of the solvent held at room temperature for several days; *M.p.* 391–392 K.

### Refinement

All H atoms were placed in calculated positions with C—H = 0.93 Å and *U*_iso_ = 1.2*U*_eq_(C). The highest residual electron density peak was located 0.14 Å from atom C19B and the deepest hole was located 0.48 Å from atom S1B.

### Crystal data

**Table:**

C_21_H_14_OS	*D*_x_ = 1.361 Mg m^−^^3^
*M**_r_* = 314.39	Melting point = 391–392 K
Orthorhombic, *P**n**a*2_1_	Mo *K*α radiation, λ = 0.71073 Å
Hall symbol: P 2c -2n	Cell parameters from 6662 reflections
*a* = 14.6675 (2) Å	θ = 1.1–27.5°
*b* = 5.5096 (1) Å	µ = 0.21 mm^−^^1^
*c* = 37.9823 (4) Å	*T* = 100 K
*V* = 3069.43 (8) Å^3^	Block, orange
*Z* = 8	0.30 × 0.12 × 0.10 mm
*F*(000) = 1312	

### Data collection

**Table:**

Bruker APEXII CCD area-detector diffractometer	6662 independent reflections
Radiation source: sealed tube	5348 reflections with *I* > 2σ(*I*)
graphite	*R*_int_ = 0.055
φ and ω scans	θ_max_ = 27.5°, θ_min_ = 1.1°
Absorption correction: multi-scan (*SADABS*; Bruker, 2005)	*h* = −19→18
*T*_min_ = 0.939, *T*_max_ = 0.979	*k* = −7→7
28929 measured reflections	*l* = −49→49

### Refinement

**Table:**

Refinement on *F*^2^	Secondary atom site location: difference Fourier map
Least-squares matrix: full	Hydrogen site location: inferred from neighbouring sites
*R*[*F*^2^ > 2σ(*F*^2^)] = 0.065	H-atom parameters constrained
*wR*(*F*^2^) = 0.200	*w* = 1/[σ^2^(*F*_o_^2^) + (0.1247*P*)^2^ + 2.1057*P*] where *P* = (*F*_o_^2^ + 2*F*_c_^2^)/3
*S* = 1.06	(Δ/σ)_max_ < 0.001
6662 reflections	Δρ_max_ = 1.58 e Å^−^^3^
391 parameters	Δρ_min_ = −0.82 e Å^−^^3^
1 restraint	Absolute structure: Flack (1983), 3093 Friedel pairs
Primary atom site location: structure-invariant direct methods	Flack parameter: 0.09 (15)

### Special details

**Table:**

Experimental. The crystal was placed in the cold stream of an Oxford Cryosystems Cobra open-flow nitrogen cryostat (Cosier & Glazer, 1986) operating at 120.0 (1) K.
Geometry. All e.s.d.'s (except the e.s.d. in the dihedral angle between two l.s. planes) are estimated using the full covariance matrix. The cell e.s.d.'s are taken into account individually in the estimation of e.s.d.'s in distances, angles and torsion angles; correlations between e.s.d.'s in cell parameters are only used when they are defined by crystal symmetry. An approximate (isotropic) treatment of cell e.s.d.'s is used for estimating e.s.d.'s involving l.s. planes.
Refinement. Refinement of *F*^2^ against ALL reflections. The weighted *R*-factor *wR* and goodness of fit *S* are based on *F*^2^, conventional *R*-factors *R* are based on *F*, with *F* set to zero for negative *F*^2^. The threshold expression of *F*^2^ > σ(*F*^2^) is used only for calculating *R*-factors(gt) *etc*. and is not relevant to the choice of reflections for refinement. *R*-factors based on *F*^2^ are statistically about twice as large as those based on *F*, and *R*- factors based on ALL data will be even larger.

### Fractional atomic coordinates and isotropic or equivalent isotropic displacement parameters (Å^2^)

**Table:**

	*x*	*y*	*z*	*U*_iso_*/*U*_eq_	
S1A	0.80198 (8)	−0.2591 (2)	0.30703 (3)	0.0253 (3)	
O1A	0.7762 (2)	−0.1293 (6)	0.38105 (8)	0.0233 (7)	
C1A	0.7530 (3)	0.1889 (8)	0.46578 (11)	0.0157 (8)	
C2A	0.8200 (3)	0.3771 (8)	0.46638 (11)	0.0201 (9)	
H2AA	0.8239	0.4840	0.4475	0.024*	
C3A	0.8784 (3)	0.4033 (9)	0.49401 (12)	0.0252 (10)	
H3AA	0.9204	0.5298	0.4942	0.030*	
C4A	0.8750 (3)	0.2366 (9)	0.52269 (13)	0.0262 (10)	
H4AA	0.9157	0.2533	0.5413	0.031*	
C5A	0.8129 (3)	0.0531 (9)	0.52310 (11)	0.0227 (9)	
H5AA	0.8121	−0.0544	0.5420	0.027*	
C6A	0.7491 (3)	0.0228 (8)	0.49507 (11)	0.0189 (9)	
C7A	0.6846 (3)	−0.1604 (8)	0.49469 (11)	0.0200 (9)	
H7AA	0.6840	−0.2712	0.5132	0.024*	
C8A	0.6203 (3)	−0.1866 (8)	0.46777 (11)	0.0176 (9)	
C9A	0.5528 (3)	−0.3732 (9)	0.46845 (13)	0.0240 (10)	
H9AA	0.5526	−0.4859	0.4867	0.029*	
C10A	0.4888 (3)	−0.3872 (9)	0.44255 (12)	0.0263 (10)	
H10A	0.4447	−0.5085	0.4433	0.032*	
C11A	0.4888 (3)	−0.2184 (10)	0.41446 (13)	0.0264 (11)	
H11A	0.4438	−0.2268	0.3973	0.032*	
C12A	0.5540 (3)	−0.0448 (8)	0.41243 (11)	0.0195 (9)	
H12A	0.5539	0.0609	0.3933	0.023*	
C13A	0.6233 (3)	−0.0193 (8)	0.43891 (10)	0.0173 (8)	
C14A	0.6914 (3)	0.1587 (8)	0.43721 (10)	0.0153 (8)	
C15A	0.6981 (3)	0.3295 (8)	0.40749 (11)	0.0186 (9)	
H15A	0.6851	0.4914	0.4123	0.022*	
C16A	0.7215 (3)	0.2743 (8)	0.37378 (12)	0.0172 (9)	
H16A	0.7196	0.3990	0.3573	0.021*	
C17A	0.7496 (3)	0.0316 (8)	0.36137 (11)	0.0169 (9)	
C18A	0.7486 (3)	−0.0075 (8)	0.32292 (11)	0.0156 (8)	
C19A	0.7103 (3)	0.1397 (9)	0.29449 (11)	0.0176 (5)	
H19A	0.6789	0.2851	0.2973	0.021*	
C20A	0.7287 (3)	0.0241 (8)	0.26182 (11)	0.0176 (5)	
H20A	0.7094	0.0859	0.2403	0.021*	
C21A	0.7784 (3)	−0.1906 (9)	0.26501 (11)	0.0176 (5)	
H21A	0.7964	−0.2851	0.2460	0.021*	
S1B	0.45484 (8)	0.2512 (2)	0.28505 (3)	0.0222 (3)	
O1B	0.4761 (2)	0.3719 (5)	0.20988 (8)	0.0224 (7)	
C1B	0.4998 (3)	0.6562 (8)	0.12502 (11)	0.0163 (8)	
C2B	0.4294 (3)	0.8335 (9)	0.12167 (11)	0.0201 (9)	
H2BA	0.4236	0.9538	0.1387	0.024*	
C3B	0.3701 (3)	0.8295 (8)	0.09376 (12)	0.0218 (9)	
H3BA	0.3256	0.9490	0.0917	0.026*	
C4B	0.3764 (3)	0.6446 (9)	0.06824 (12)	0.0225 (9)	
H4BA	0.3353	0.6422	0.0496	0.027*	
C5B	0.4412 (3)	0.4706 (8)	0.07042 (11)	0.0198 (9)	
H5BA	0.4435	0.3491	0.0534	0.024*	
C6B	0.5066 (3)	0.4707 (8)	0.09854 (10)	0.0152 (8)	
C7B	0.5750 (3)	0.2983 (8)	0.10025 (11)	0.0181 (9)	
H7BA	0.5776	0.1762	0.0834	0.022*	
C8B	0.6408 (3)	0.3054 (8)	0.12722 (11)	0.0174 (9)	
C9B	0.7149 (3)	0.1350 (8)	0.12850 (12)	0.0217 (9)	
H9BA	0.7190	0.0142	0.1115	0.026*	
C10B	0.7797 (3)	0.1477 (9)	0.15438 (13)	0.0268 (10)	
H10B	0.8272	0.0361	0.1548	0.032*	
C11B	0.7745 (3)	0.3299 (9)	0.18054 (12)	0.0230 (10)	
H11B	0.8194	0.3384	0.1978	0.028*	
C12B	0.7041 (3)	0.4945 (9)	0.18086 (12)	0.0198 (9)	
H12B	0.7016	0.6119	0.1984	0.024*	
C13B	0.6343 (3)	0.4872 (8)	0.15435 (10)	0.0157 (8)	
C14B	0.5611 (3)	0.6550 (8)	0.15328 (11)	0.0150 (8)	
C15B	0.5492 (3)	0.8349 (8)	0.18238 (12)	0.0181 (9)	
H15B	0.5587	0.9976	0.1770	0.022*	
C16B	0.5261 (3)	0.7810 (8)	0.21535 (12)	0.0169 (9)	
H16B	0.5247	0.9086	0.2314	0.020*	
C17B	0.5024 (3)	0.5369 (8)	0.22900 (11)	0.0163 (8)	
C18B	0.5063 (3)	0.5004 (8)	0.26765 (12)	0.0180 (8)	
C19B	0.5461 (3)	0.6449 (9)	0.29485 (11)	0.0186 (5)	
H19B	0.5774	0.7895	0.2911	0.022*	
C20B	0.5319 (3)	0.5402 (8)	0.32761 (11)	0.0186 (5)	
H20B	0.5538	0.6075	0.3484	0.022*	
C21B	0.4834 (3)	0.3311 (9)	0.32663 (11)	0.0186 (5)	
H21B	0.4675	0.2420	0.3465	0.022*	

### Atomic displacement parameters (Å^2^)

**Table:**

	*U*^11^	*U*^22^	*U*^33^	*U*^12^	*U*^13^	*U*^23^
S1A	0.0264 (6)	0.0243 (7)	0.0251 (6)	−0.0017 (5)	0.0031 (5)	−0.0036 (5)
O1A	0.0312 (17)	0.0193 (17)	0.0194 (14)	0.0059 (14)	0.0025 (13)	0.0055 (14)
C1A	0.0149 (19)	0.016 (2)	0.0165 (19)	0.0051 (16)	0.0006 (15)	−0.0002 (18)
C2A	0.022 (2)	0.019 (2)	0.0196 (19)	0.0016 (17)	0.0055 (16)	0.0007 (19)
C3A	0.021 (2)	0.025 (2)	0.030 (2)	−0.0011 (18)	−0.0034 (18)	−0.006 (2)
C4A	0.024 (2)	0.030 (3)	0.024 (2)	0.0028 (19)	−0.0063 (18)	−0.008 (2)
C5A	0.025 (2)	0.027 (2)	0.0158 (18)	0.0093 (19)	−0.0007 (17)	−0.0011 (19)
C6A	0.021 (2)	0.021 (2)	0.0150 (18)	0.0088 (17)	0.0036 (16)	−0.0006 (18)
C7A	0.023 (2)	0.021 (2)	0.0162 (19)	0.0065 (18)	0.0027 (16)	0.0061 (19)
C8A	0.0172 (19)	0.015 (2)	0.021 (2)	0.0031 (16)	0.0082 (16)	−0.0008 (19)
C9A	0.026 (2)	0.017 (2)	0.029 (2)	−0.0006 (17)	0.0103 (18)	0.002 (2)
C10A	0.026 (2)	0.023 (2)	0.030 (2)	−0.0091 (19)	0.0108 (18)	−0.006 (2)
C11A	0.018 (2)	0.036 (3)	0.025 (2)	0.0000 (19)	−0.0004 (17)	−0.010 (2)
C12A	0.024 (2)	0.020 (2)	0.0147 (19)	0.0038 (17)	0.0001 (16)	0.0000 (19)
C13A	0.019 (2)	0.019 (2)	0.0134 (18)	0.0019 (17)	−0.0010 (15)	−0.0045 (18)
C14A	0.023 (2)	0.013 (2)	0.0103 (18)	0.0027 (16)	0.0032 (15)	−0.0018 (17)
C15A	0.027 (2)	0.013 (2)	0.016 (2)	−0.0008 (16)	0.0002 (16)	−0.0055 (19)
C16A	0.022 (2)	0.014 (2)	0.016 (2)	0.0029 (17)	−0.0010 (18)	0.0068 (17)
C17A	0.0174 (19)	0.014 (2)	0.019 (2)	−0.0061 (16)	0.0048 (15)	0.0018 (18)
C18A	0.0151 (18)	0.016 (2)	0.0156 (17)	−0.0043 (15)	0.0055 (15)	−0.0018 (17)
C19A	0.0125 (11)	0.0246 (13)	0.0158 (11)	−0.0094 (10)	0.0020 (9)	−0.0044 (11)
C20A	0.0125 (11)	0.0246 (13)	0.0158 (11)	−0.0094 (10)	0.0020 (9)	−0.0044 (11)
C21A	0.0125 (11)	0.0246 (13)	0.0158 (11)	−0.0094 (10)	0.0020 (9)	−0.0044 (11)
S1B	0.0233 (6)	0.0204 (6)	0.0230 (6)	0.0010 (4)	0.0023 (4)	0.0046 (5)
O1B	0.0306 (17)	0.0165 (16)	0.0200 (14)	−0.0019 (13)	0.0020 (13)	−0.0011 (14)
C1B	0.019 (2)	0.014 (2)	0.0161 (19)	0.0011 (16)	0.0065 (15)	0.0049 (18)
C2B	0.022 (2)	0.022 (2)	0.0166 (19)	0.0012 (18)	0.0014 (16)	0.0026 (18)
C3B	0.019 (2)	0.020 (2)	0.026 (2)	0.0002 (17)	−0.0013 (17)	0.007 (2)
C4B	0.020 (2)	0.028 (3)	0.0185 (19)	−0.0049 (19)	−0.0027 (16)	0.001 (2)
C5B	0.024 (2)	0.021 (2)	0.0141 (18)	−0.0022 (17)	−0.0021 (16)	0.0000 (18)
C6B	0.0152 (19)	0.018 (2)	0.0122 (17)	−0.0054 (15)	0.0011 (15)	−0.0010 (17)
C7B	0.021 (2)	0.020 (2)	0.0133 (18)	−0.0024 (17)	0.0062 (16)	−0.0033 (18)
C8B	0.019 (2)	0.017 (2)	0.016 (2)	0.0006 (17)	0.0050 (16)	0.0041 (18)
C9B	0.020 (2)	0.019 (2)	0.026 (2)	0.0022 (17)	0.0098 (17)	−0.001 (2)
C10B	0.024 (2)	0.026 (3)	0.031 (2)	0.007 (2)	0.0083 (19)	0.008 (2)
C11B	0.020 (2)	0.028 (3)	0.022 (2)	0.0023 (19)	0.0002 (17)	0.009 (2)
C12B	0.020 (2)	0.021 (2)	0.019 (2)	0.0012 (17)	−0.0006 (16)	0.0012 (18)
C13B	0.0178 (19)	0.014 (2)	0.0151 (18)	0.0009 (16)	0.0059 (15)	0.0005 (18)
C14B	0.0174 (19)	0.012 (2)	0.0160 (18)	−0.0009 (16)	0.0016 (15)	0.0015 (18)
C15B	0.0163 (19)	0.014 (2)	0.024 (2)	−0.0019 (15)	0.0031 (16)	−0.002 (2)
C16B	0.023 (2)	0.014 (2)	0.014 (2)	−0.0007 (17)	0.0004 (18)	−0.0044 (17)
C17B	0.0171 (19)	0.017 (2)	0.0147 (18)	−0.0021 (16)	0.0040 (15)	−0.0011 (18)
C18B	0.0157 (18)	0.017 (2)	0.021 (2)	0.0030 (16)	0.0051 (16)	−0.0023 (18)
C19B	0.0145 (11)	0.0243 (14)	0.0171 (11)	0.0086 (10)	0.0017 (9)	0.0021 (11)
C20B	0.0145 (11)	0.0243 (14)	0.0171 (11)	0.0086 (10)	0.0017 (9)	0.0021 (11)
C21B	0.0145 (11)	0.0243 (14)	0.0171 (11)	0.0086 (10)	0.0017 (9)	0.0021 (11)

### Geometric parameters (Å, °)

**Table:**

S1A—C21A	1.676 (4)	S1B—C21B	1.692 (5)
S1A—C18A	1.703 (4)	S1B—C18B	1.700 (5)
O1A—C17A	1.224 (5)	O1B—C17B	1.226 (5)
C1A—C14A	1.422 (6)	C1B—C14B	1.400 (6)
C1A—C2A	1.430 (6)	C1B—C2B	1.427 (6)
C1A—C6A	1.442 (6)	C1B—C6B	1.438 (6)
C2A—C3A	1.362 (6)	C2B—C3B	1.372 (6)
C2A—H2AA	0.9300	C2B—H2BA	0.9300
C3A—C4A	1.426 (7)	C3B—C4B	1.409 (7)
C3A—H3AA	0.9300	C3B—H3BA	0.9300
C4A—C5A	1.361 (7)	C4B—C5B	1.353 (6)
C4A—H4AA	0.9300	C4B—H4BA	0.9300
C5A—C6A	1.428 (6)	C5B—C6B	1.435 (5)
C5A—H5AA	0.9300	C5B—H5BA	0.9300
C6A—C7A	1.383 (6)	C6B—C7B	1.383 (6)
C7A—C8A	1.398 (6)	C7B—C8B	1.408 (6)
C7A—H7AA	0.9300	C7B—H7BA	0.9300
C8A—C9A	1.427 (6)	C8B—C9B	1.437 (6)
C8A—C13A	1.433 (6)	C8B—C13B	1.440 (6)
C9A—C10A	1.362 (7)	C9B—C10B	1.368 (7)
C9A—H9AA	0.9300	C9B—H9BA	0.9300
C10A—C11A	1.415 (7)	C10B—C11B	1.415 (7)
C10A—H10A	0.9300	C10B—H10B	0.9300
C11A—C12A	1.354 (7)	C11B—C12B	1.374 (6)
C11A—H11A	0.9300	C11B—H11B	0.9300
C12A—C13A	1.437 (5)	C12B—C13B	1.436 (5)
C12A—H12A	0.9300	C12B—H12B	0.9300
C13A—C14A	1.401 (6)	C13B—C14B	1.417 (6)
C14A—C15A	1.473 (6)	C14B—C15B	1.495 (6)
C15A—C16A	1.360 (6)	C15B—C16B	1.331 (6)
C15A—H15A	0.9300	C15B—H15B	0.9300
C16A—C17A	1.477 (6)	C16B—C17B	1.483 (6)
C16A—H16A	0.9300	C16B—H16B	0.9300
C17A—C18A	1.476 (5)	C17B—C18B	1.483 (6)
C18A—C19A	1.463 (6)	C18B—C19B	1.430 (6)
C19A—C20A	1.421 (6)	C19B—C20B	1.387 (6)
C19A—H19A	0.9300	C19B—H19B	0.9300
C20A—C21A	1.395 (6)	C20B—C21B	1.355 (7)
C20A—H20A	0.9300	C20B—H20B	0.9300
C21A—H21A	0.9300	C21B—H21B	0.9300
			
C21A—S1A—C18A	93.4 (2)	C21B—S1B—C18B	92.5 (2)
C14A—C1A—C2A	122.3 (4)	C14B—C1B—C2B	122.4 (4)
C14A—C1A—C6A	119.3 (4)	C14B—C1B—C6B	119.2 (4)
C2A—C1A—C6A	118.4 (4)	C2B—C1B—C6B	118.3 (4)
C3A—C2A—C1A	121.5 (4)	C3B—C2B—C1B	121.1 (4)
C3A—C2A—H2AA	119.3	C3B—C2B—H2BA	119.4
C1A—C2A—H2AA	119.3	C1B—C2B—H2BA	119.4
C2A—C3A—C4A	119.9 (4)	C2B—C3B—C4B	120.1 (4)
C2A—C3A—H3AA	120.1	C2B—C3B—H3BA	120.0
C4A—C3A—H3AA	120.1	C4B—C3B—H3BA	120.0
C5A—C4A—C3A	120.7 (4)	C5B—C4B—C3B	121.1 (4)
C5A—C4A—H4AA	119.6	C5B—C4B—H4BA	119.4
C3A—C4A—H4AA	119.6	C3B—C4B—H4BA	119.4
C4A—C5A—C6A	121.2 (4)	C4B—C5B—C6B	121.0 (4)
C4A—C5A—H5AA	119.4	C4B—C5B—H5BA	119.5
C6A—C5A—H5AA	119.4	C6B—C5B—H5BA	119.5
C7A—C6A—C5A	122.8 (4)	C7B—C6B—C5B	121.3 (4)
C7A—C6A—C1A	118.8 (4)	C7B—C6B—C1B	120.4 (4)
C5A—C6A—C1A	118.4 (4)	C5B—C6B—C1B	118.3 (4)
C6A—C7A—C8A	122.9 (4)	C6B—C7B—C8B	120.8 (4)
C6A—C7A—H7AA	118.5	C6B—C7B—H7BA	119.6
C8A—C7A—H7AA	118.5	C8B—C7B—H7BA	119.6
C7A—C8A—C9A	121.9 (4)	C7B—C8B—C9B	121.7 (4)
C7A—C8A—C13A	118.2 (4)	C7B—C8B—C13B	119.6 (4)
C9A—C8A—C13A	119.9 (4)	C9B—C8B—C13B	118.7 (4)
C10A—C9A—C8A	120.4 (4)	C10B—C9B—C8B	121.1 (4)
C10A—C9A—H9AA	119.8	C10B—C9B—H9BA	119.5
C8A—C9A—H9AA	119.8	C8B—C9B—H9BA	119.5
C9A—C10A—C11A	120.5 (4)	C9B—C10B—C11B	120.2 (4)
C9A—C10A—H10A	119.8	C9B—C10B—H10B	119.9
C11A—C10A—H10A	119.8	C11B—C10B—H10B	119.9
C12A—C11A—C10A	120.5 (4)	C12B—C11B—C10B	121.0 (4)
C12A—C11A—H11A	119.8	C12B—C11B—H11B	119.5
C10A—C11A—H11A	119.8	C10B—C11B—H11B	119.5
C11A—C12A—C13A	121.9 (4)	C11B—C12B—C13B	120.7 (4)
C11A—C12A—H12A	119.0	C11B—C12B—H12B	119.6
C13A—C12A—H12A	119.0	C13B—C12B—H12B	119.6
C14A—C13A—C8A	120.5 (4)	C14B—C13B—C12B	122.8 (4)
C14A—C13A—C12A	122.7 (4)	C14B—C13B—C8B	118.9 (4)
C8A—C13A—C12A	116.8 (4)	C12B—C13B—C8B	118.3 (4)
C13A—C14A—C1A	120.0 (4)	C1B—C14B—C13B	120.8 (4)
C13A—C14A—C15A	122.0 (4)	C1B—C14B—C15B	119.2 (4)
C1A—C14A—C15A	117.9 (4)	C13B—C14B—C15B	120.0 (4)
C16A—C15A—C14A	126.6 (4)	C16B—C15B—C14B	125.3 (4)
C16A—C15A—H15A	116.7	C16B—C15B—H15B	117.4
C14A—C15A—H15A	116.7	C14B—C15B—H15B	117.4
C15A—C16A—C17A	125.0 (4)	C15B—C16B—C17B	126.2 (4)
C15A—C16A—H16A	117.5	C15B—C16B—H16B	116.9
C17A—C16A—H16A	117.5	C17B—C16B—H16B	116.9
O1A—C17A—C18A	120.1 (4)	O1B—C17B—C18B	119.9 (4)
O1A—C17A—C16A	123.4 (4)	O1B—C17B—C16B	122.6 (4)
C18A—C17A—C16A	116.4 (4)	C18B—C17B—C16B	117.4 (4)
C19A—C18A—C17A	130.8 (4)	C19B—C18B—C17B	131.0 (4)
C19A—C18A—S1A	111.5 (3)	C19B—C18B—S1B	110.5 (3)
C17A—C18A—S1A	117.7 (3)	C17B—C18B—S1B	118.5 (3)
C20A—C19A—C18A	108.9 (4)	C20B—C19B—C18B	110.8 (4)
C20A—C19A—H19A	125.6	C20B—C19B—H19B	124.6
C18A—C19A—H19A	125.6	C18B—C19B—H19B	124.6
C21A—C20A—C19A	113.8 (4)	C21B—C20B—C19B	114.1 (4)
C21A—C20A—H20A	123.1	C21B—C20B—H20B	123.0
C19A—C20A—H20A	123.1	C19B—C20B—H20B	123.0
C20A—C21A—S1A	112.4 (3)	C20B—C21B—S1B	112.1 (3)
C20A—C21A—H21A	123.8	C20B—C21B—H21B	123.9
S1A—C21A—H21A	123.8	S1B—C21B—H21B	123.9
			
C14A—C1A—C2A—C3A	179.9 (4)	C14B—C1B—C2B—C3B	−179.7 (4)
C6A—C1A—C2A—C3A	−0.9 (6)	C6B—C1B—C2B—C3B	−0.7 (6)
C1A—C2A—C3A—C4A	1.8 (7)	C1B—C2B—C3B—C4B	1.7 (7)
C2A—C3A—C4A—C5A	−1.1 (7)	C2B—C3B—C4B—C5B	−0.9 (7)
C3A—C4A—C5A—C6A	−0.4 (7)	C3B—C4B—C5B—C6B	−0.9 (7)
C4A—C5A—C6A—C7A	−179.6 (4)	C4B—C5B—C6B—C7B	−177.7 (4)
C4A—C5A—C6A—C1A	1.2 (6)	C4B—C5B—C6B—C1B	1.9 (6)
C14A—C1A—C6A—C7A	−0.7 (6)	C14B—C1B—C6B—C7B	−2.4 (6)
C2A—C1A—C6A—C7A	−179.8 (4)	C2B—C1B—C6B—C7B	178.5 (4)
C14A—C1A—C6A—C5A	178.6 (4)	C14B—C1B—C6B—C5B	178.0 (4)
C2A—C1A—C6A—C5A	−0.6 (6)	C2B—C1B—C6B—C5B	−1.1 (6)
C5A—C6A—C7A—C8A	178.2 (4)	C5B—C6B—C7B—C8B	177.6 (4)
C1A—C6A—C7A—C8A	−2.6 (6)	C1B—C6B—C7B—C8B	−2.0 (6)
C6A—C7A—C8A—C9A	−178.3 (4)	C6B—C7B—C8B—C9B	−177.0 (4)
C6A—C7A—C8A—C13A	1.7 (6)	C6B—C7B—C8B—C13B	3.0 (6)
C7A—C8A—C9A—C10A	177.2 (4)	C7B—C8B—C9B—C10B	178.4 (4)
C13A—C8A—C9A—C10A	−2.7 (6)	C13B—C8B—C9B—C10B	−1.6 (6)
C8A—C9A—C10A—C11A	0.6 (7)	C8B—C9B—C10B—C11B	0.1 (7)
C9A—C10A—C11A—C12A	1.8 (7)	C9B—C10B—C11B—C12B	1.0 (7)
C10A—C11A—C12A—C13A	−2.1 (7)	C10B—C11B—C12B—C13B	−0.6 (7)
C7A—C8A—C13A—C14A	2.6 (6)	C11B—C12B—C13B—C14B	−179.1 (4)
C9A—C8A—C13A—C14A	−177.4 (4)	C11B—C12B—C13B—C8B	−0.8 (6)
C7A—C8A—C13A—C12A	−177.5 (4)	C7B—C8B—C13B—C14B	0.3 (6)
C9A—C8A—C13A—C12A	2.4 (6)	C9B—C8B—C13B—C14B	−179.7 (4)
C11A—C12A—C13A—C14A	179.8 (4)	C7B—C8B—C13B—C12B	−178.1 (4)
C11A—C12A—C13A—C8A	−0.1 (6)	C9B—C8B—C13B—C12B	1.9 (6)
C8A—C13A—C14A—C1A	−5.9 (6)	C2B—C1B—C14B—C13B	−175.3 (4)
C12A—C13A—C14A—C1A	174.3 (4)	C6B—C1B—C14B—C13B	5.7 (6)
C8A—C13A—C14A—C15A	178.3 (4)	C2B—C1B—C14B—C15B	4.2 (6)
C12A—C13A—C14A—C15A	−1.5 (6)	C6B—C1B—C14B—C15B	−174.8 (4)
C2A—C1A—C14A—C13A	−176.0 (4)	C12B—C13B—C14B—C1B	173.6 (4)
C6A—C1A—C14A—C13A	4.8 (6)	C8B—C13B—C14B—C1B	−4.7 (6)
C2A—C1A—C14A—C15A	0.0 (6)	C12B—C13B—C14B—C15B	−5.9 (6)
C6A—C1A—C14A—C15A	−179.2 (4)	C8B—C13B—C14B—C15B	175.9 (4)
C13A—C14A—C15A—C16A	−68.5 (6)	C1B—C14B—C15B—C16B	112.9 (5)
C1A—C14A—C15A—C16A	115.5 (5)	C13B—C14B—C15B—C16B	−67.7 (6)
C14A—C15A—C16A—C17A	−3.7 (7)	C14B—C15B—C16B—C17B	−4.0 (7)
C15A—C16A—C17A—O1A	−18.5 (7)	C15B—C16B—C17B—O1B	−21.8 (7)
C15A—C16A—C17A—C18A	164.3 (4)	C15B—C16B—C17B—C18B	161.7 (4)
O1A—C17A—C18A—C19A	171.3 (4)	O1B—C17B—C18B—C19B	169.3 (4)
C16A—C17A—C18A—C19A	−11.4 (6)	C16B—C17B—C18B—C19B	−14.0 (7)
O1A—C17A—C18A—S1A	−10.4 (5)	O1B—C17B—C18B—S1B	−11.8 (5)
C16A—C17A—C18A—S1A	166.9 (3)	C16B—C17B—C18B—S1B	164.8 (3)
C21A—S1A—C18A—C19A	−0.1 (3)	C21B—S1B—C18B—C19B	0.3 (3)
C21A—S1A—C18A—C17A	−178.7 (3)	C21B—S1B—C18B—C17B	−178.8 (3)
C17A—C18A—C19A—C20A	179.1 (4)	C17B—C18B—C19B—C20B	179.2 (4)
S1A—C18A—C19A—C20A	0.7 (4)	S1B—C18B—C19B—C20B	0.3 (4)
C18A—C19A—C20A—C21A	−1.1 (5)	C18B—C19B—C20B—C21B	−1.0 (5)
C19A—C20A—C21A—S1A	1.0 (4)	C19B—C20B—C21B—S1B	1.2 (5)
C18A—S1A—C21A—C20A	−0.5 (3)	C18B—S1B—C21B—C20B	−0.8 (3)

### Hydrogen-bond geometry (Å, °)

**Table:**

*D*—H···*A*	*D*—H	H···*A*	*D*···*A*	*D*—H···*A*
C3A—H3AA···Cg3^i^	0.93	2.99	3.679 (5)	132
C10A—H10A···Cg2^ii^	0.93	2.95	3.694 (5)	138
C10B—H10B···Cg5^i^	0.93	2.93	3.594 (5)	129
C15A—H15A···Cg3^iii^	0.93	2.76	3.550 (5)	143
C15B—H15B···Cg6^iii^	0.93	2.94	3.689 (5)	139
C19A—H19A···Cg4	0.93	2.72	3.486 (5)	140
C19B—H19B···Cg1^iii^	0.93	2.72	3.458 (5)	137

Symmetry codes: (i) *x*+1/2, −*y*+1/2, *z*; (ii) *x*−1/2, −*y*−1/2, *z*; (iii) *x*, *y*+1, *z*.
